# Novel use of cranial epidural space in rabbits as an animal model to investigate bone volume augmentation potential of different bone graft substitutes

**DOI:** 10.1186/s13005-016-0131-z

**Published:** 2016-12-01

**Authors:** Ivan Valdivia-Gandur, Wilfried Engelke, Víctor Beltrán, Eduardo Borie, Ramón Fuentes, María Cristina Manzanares-Céspedes

**Affiliations:** 1 Biomedical Department, Universidad de Antofagasta, Antofagasta, Chile; 2 Odontology Department, Universidad de Antofagasta, Antofagasta, Chile; 3 Department of Oral and Maxillofacial Surgery, Georg-August-Universität, Göttingen, Germany; 4 Faculty of Dentistry, Universidad de La Frontera, Temuco, Chile; 5 Muscular and Skeletal Pathology Research, Human Anatomy and Embryology Unit, Universitat de Barcelona, Barcelona, Spain; 6 Clinical Investigation and Dental Innovation Center (CIDIC), Dental School, Universidad de La Frontera, Av. Francisco Salazar 01145, Temuco, Chile

**Keywords:** Bone augmentation, BCP, Bi-phasic calcium phosphate, Beta-tricalcium phosphate, Epidural space, Hydroxyapatite, Rabbit, Sinus floor elevation

## Abstract

**Background:**

The success of bone augmentation to a major degree depends on the biomechanics and biological conditions of the surrounding tissues. Therefore, an animal model is needed providing anatomical sites with similar mechanical pressures for comparing its influence on different biomaterials for bone regeneration. The present report describes the new bone formation associated to biomaterial in a bursa created in the epidural space, between dura mater and cranial calvaria, under the constant pressure of cerebrospinal fluid.

**Methods:**

Five adult California rabbits were used for the trial. In each animal, two bursae were created in the epidural spaces, in the anterior part of the skull, below both sides of the interfrontal suture. The spaces between dura mater and cranial calvaria were filled with *in-situ* hardening biphasic calcium phosphate containing hydroxyapatite and beta tricalcium-phosphate (BCP), *in-situ* hardening phase-pure beta-tricalcium phosphate (β-TCP) or without any biomaterials (sham). After 90 days, the animals were sacrificed, and the defect sites were extracted and processed for histomorphometric analysis by optical and backscattered electron microscopy.

**Results:**

The cranial epidural spaces created (*n* = 10) could be preserved by the application both BCP (*n* = 3) and β-TCP biomaterials (*n* = 3) in all experimental sites. The sites augmented with BCP showed less new bone formation but a trend to better volume preservation than the sites augmented with β-TCP. However, the bone in the BCP sites seemed to be more mature as indicated by the higher percentage of lamellar bone in the sites. In contrast, the created space could not be preserved, and new bone formation was scarce in the sham-operated sites (*n* = 4).

**Conclusion:**

The experimental bursae created bilaterally in the epidural space allows comparing objectively bone formation in relation to biomaterials for bone regeneration under permanent physiological forces from cerebrospinal fluid pressure.

## Background

Ideally, a biomaterial for bone regeneration should have osteoconductive and osteoinductive properties. In addition, the biomaterial must maintain its dimensional stability for a certain period in the defect site where is applied to preserve the augmented volume and to allow bone growth and the biomaterial osteointegration, even under physiological pressures. This factor is especially important when the biomaterials are applied to increase the bone volume for example in the case of the maxillary sinus floor elevation or alveolar crest augmentation, when the objective is to place dental implants [1, 2]. In order to investigate these important features several in vivo models [1, 3–5] have been developed.

Due to the presence of the dura mater and its periosteal layer that facilitates bone regeneration, the skullcap is an anatomic site frequently used for studies concerning biomaterials by means of critical size defects in various animal models [6–10]. The dura mater overlying the inner surface of the cranial vault has been shown to have osteogenerative potential due to its osteogenic layer [11, 12]. Furthermore, it has been described that its integrity is important for bone regeneration in experimental skull calvarial defects [13, 14] in which the biomaterial is placed in the bone defect. However, in the literature, the cranial epidural space itself seems to be disregarded as an anatomical site for bone regeneration studies. In this anatomical site, biomaterial particles could be inserted in bursae atraumatically opened between the dura mater and the cranial vault surface at a distance of the bone defect created for accessing. This model may be an advantage to analyse if a specific biomaterial can maintain the space created being subjected to the constant hydrodynamic pressure of cerebrospinal fluid, which is transmitted through the dura mater to the biomaterial.

Therefore, the aim of this research is to show the first results in the use of bilateral bursae created in the epidural space of small animal models, for testing bone regeneration associated to biomaterials under the constant hydrodynamic pressure of cerebrospinal fluid.

## Methods

**Fig. 1:**
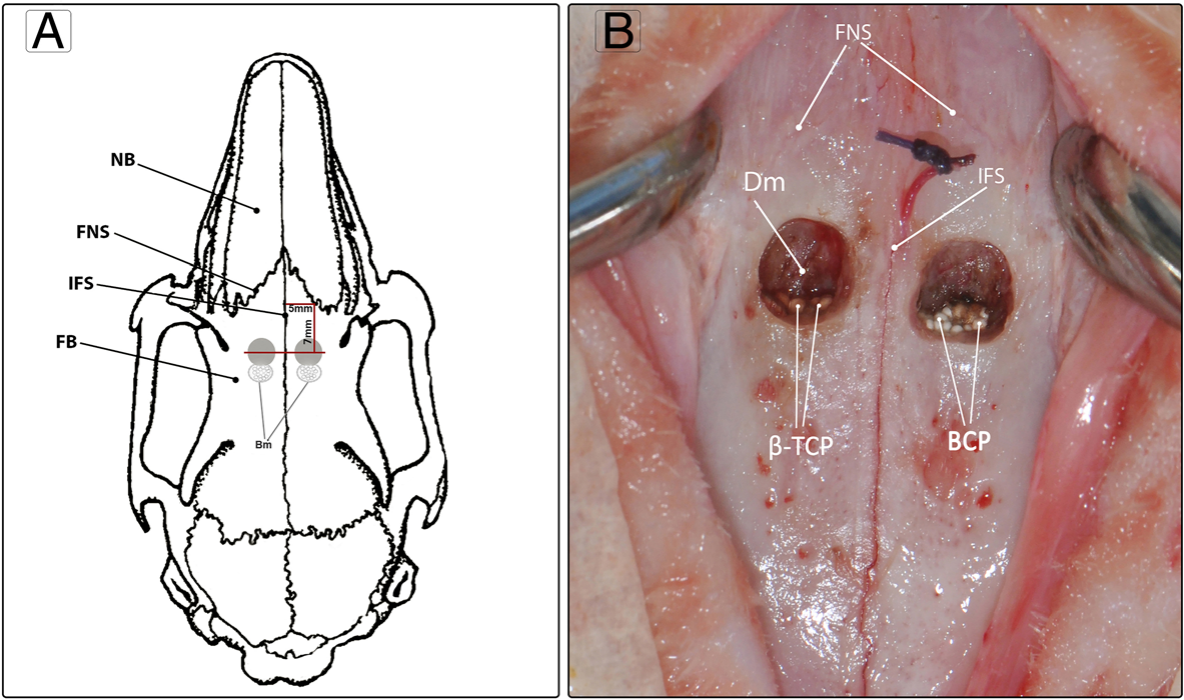
Biomaterial placement in the experimental epidural space. **a**: Schematic view of a rabbit skull, grey circles represent the drilling sites in the frontal bone and the grey-dotted circles the “bursae” created for the epidural insertion of the biomaterial. Red lines represent the spatial localization of perforations and biomaterial placement. **b**: Perforation of the cortical plate and biomaterial placement inside epidural spaces generated. Bm: site for biomaterial placement inside cranial epidural space created. Dm: dura mater FB: frontal bone, NB: nasal bone; IFS: interfrontal suture; FNS: fronto nasal suture; BCP and β-TCP: Biomaterials (see detailed description in the text) inside the bursae in the epidural cranial space

The protocol was approved by the Ethics Committee of the Universidad de La Frontera. Five adult California rabbits were used. As experimental sites, epidural spaces were created below both sides of the interfrontal suture in the anterior zone of the skull (Fig. 1).

### Surgical model

The animals were kept under regulated conditions with a standard laboratory diet. General anaesthesia was induced by intramuscular injections of Ketamine 30 mg/kg and Xylazine 5 mg/kg supplemented with intraoperative analgesia by subcutaneous injection of Buprenorphine 0.3 ml/kg at 30-min intervals during surgery. Furthermore, local anaesthesia of 0.4 ml Lidocaine 2% with epinephrine 1:50,000 was applied. Preoperative shaving was done, and the surface was cleaned carefully with iodized alcohol. The excess was removed with sterile gauze. Surgical access was performed via an anteroposterior skin incision in the fronto-parietal region. Bleeding vessels were ligated when necessary. The osseous accesses were prepared using a round surgical bur with 3 mm of diameter, and subsequently, bilateral epidural spaces (“bursae”) of about 0.15 cm^3^ were created towards the dorsal direction by atraumatic separation of dura mater from the inner bone surface. The osseous access sites to create epidural spaces were circular, and its centers were established 7 mm in the dorsal direction from a point located in the nasofrontal suture and 5 mm lateral respect to the interfrontal suture (Fig. 1a). The *bursae* were created by separating the duramater from the osseous surface dorsal to the accesses with a series of small size osteotomes. Particular cares were taken for avoiding damage to emissary venous vessels or dura mater perforation. To obtain spaces with similar size, the same atraumatic tool was used on both sides (Fig. 1b). The biomaterials inserted in the spaces created were a resorbable *in-situ* hardening phase-pure beta-tricalcium phosphate (β-TCP – GUIDOR easy-graft CLASSIC, Sunstar Suisse SA, Etoy, Switzerland) and a partly resorbable *in-situ* hardening cement of biphasic calcium phosphate, 60% hydroxyapatite and 40% β-TCP (BCP - GUIDOR easy-graft CRYSTAL, Sunstar Suisse SA, Etoy, Switzerland). These biomaterials are applied as a homogenous mouldable mass and do not harden until in contact with body fluids. In animal #1, BCP was placed inside the epidural space created in the right side, leaving the contralateral side empty as control (sham operation). In animal #2, the same process was performed, but the biomaterial used was β-TCP. In animals #3 and #4, BCP was inserted in the right side cavity and β-TCP in the contralateral side (Fig. 1b). In animal #5, both bilateral epidural spaces without placing biomaterial were created as sham operation. A volume of 0.15 cm^3^ of biomaterial was used for all experimental sites. In the postoperatory, antibiotherapy by oxytetracycline (1 ml/kg subcutaneously every 12 h for three days) and analgesia using meloxicam (0.2 mg/Kg once daily for three days) were administered to each animal. The postoperative period was controlled using protocols of Morton and Griffiths [15] and Southwestern Medical Center IACUC [16] to recognize stress and discomfort in experimental animals. After a period of 90 days for healing, the animals were euthanized with pentobarbital overdose. Subsequently, anatomical segments of skullcap were extracted and fixed for at least 48 h in 3.7% formaldehyde at 5 °C. Posteriorly, the segments were reduced to obtain approximately 3 × 3 cm of sample size, following the anatomical references used for osseous access to prevent damage in the target area. Then, the segments were processed for inclusion in resin (Technovit 9100) for histomorphometric analysis.

### Histomorphometric analysis

**Fig. 2:**
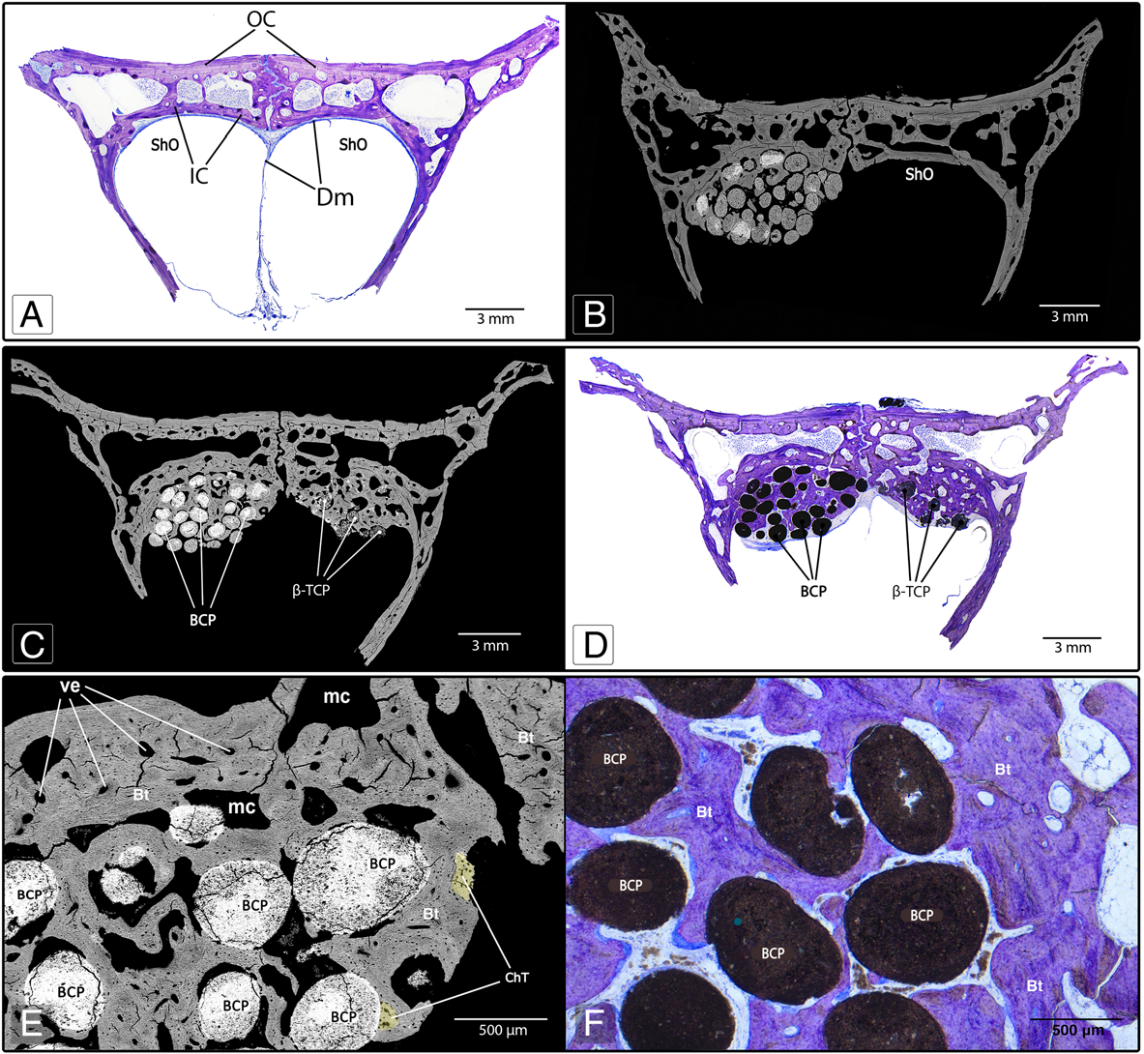
Frontal cut of samples from rabbit calvaria used in the experimental protocol. **a**: Control specimen without biomaterial placement (sham operated) where it is observed that the epidural space experimentally created is not maintained. **b**: Experimental specimen with the unilateral insertion of BCP. **c** and **d**: Specimen with the bilateral insertion of BCP and β-TCP. **e** and **f**: Integration of the hydroxyapatite particles within the bone tissue. At 90 days, the epidural space created was preserved with the biomaterial inserted. Significant osteoconductive capacity was observed for both biomaterials. New mature trabecular bone is highly vascularized with small sections of chondroid tissue (image **c**, highlighted in yellow). **b**, **c** and **e**: Backscatter electron microscopy; **a**, **d** and **f**: Toluidine blue histology; OC: outer cortex; IC: inner cortex; Dm: dura mater; ShO: Sham operated area; ve: vascular spaces, mc: medullary cavity; Bt: bone tissue; ChT: Chondroid tissue

The segments included in the resin were subjected to frontal cuts every two millimetres in the dorsal direction perpendicularly to the interfrontal suture using a thin circular saw, starting from the anatomical sites where osseous access was created (Fig. 1). The three central sections containing the biomaterial and associated bone tissue were treated for study using backscattered electron microscopy and optic microscopy. For the measurement, standardized photographs of these samples with 75× magnification were obtained using backscatter electron microscopy (BS-SEM) and INCA software (Oxford instruments). Posteriorly, photographs were assembled to form a complete picture for each sample with Adobe Photoshop CC software. For identification of tissue and area quantification, the photographic analysis of samples was conducted as described by Goret-Nicaise et al. [17] and Manzanares et al. [18], in which old bone tissue areas, newly formed bone, fibroreticular bone tissue, lamellar bone, chondroid tissue, and vascular spaces are identified by images obtained with BS-SEM. Measurements of the areas at the assembled photographs were performed using the software “Image J” (http://imagej.nih.gov/ij/). Bone tissue area was calculated as percentage values of the “total observed area”, defined as the quantity of bone tissue, particles of biomaterial, and spaces observed in the treated zone (Fig. 2). Percentages of the various types of tissue observed were calculated considering the total bone area present in the sample studied. Furthermore, vascular and marrow spaces were calculated as percentages of the total observed area of the sample studied (Table 1). Posteriorly, the samples included in resin were processed by wear and polish to obtain a thin sheet of 30–40 microns thickness, which were prepared and stained with toluidine blue. Statgraphics Sigma® was used as software for statistic analysis of data. Additional information was obtained by optical microscopy analysis using toluidine blue on resin-included samples.

**Table 1:** Distribution of bone tissue observed inside experimental cranial epidural space identified by BS-SEM [17, 18]

Tissue		Sample with β-TCP	Sample with BCP
Bone tissue/Total observed area × 100		63.6% +/− 8.7 *	48.5% +/− 9.4
Vascular and Medullary cavity/Total observed area × 100		29.5% +/− 8.4	38.2% +/− 7.2
Characteristics of bone tissue	Chondroid/tissue Total bone area × 100	5.1% +/− 2.2	3.1% +/− 3
	Woven bone/Total bone area × 100	19.3% +/− 6.2	16.4% +/− 8.1
	Lamellar bone/Total bone area × 100	78.4% +/− 9.5	85.7% +/− 13.3

Percentage distribution of tissues observed in the experimental samples by BS-SEM. The “total observed area” involves all of tissue observed in the epidural space including biomaterial particles, in a frontal cut of sample (as showed in Fig. 2). The “total bone area” involves only the bone tissue (without biomaterial particles) observed in a frontal cut of samples. BCP samples reveal a more mature profile of osseous tissue than β-TCP with higher amounts of lamellar bone, albeit the difference is not significant

*Significant difference (*p*<0,05)

## Results

Weight and behaviour of the animals were normal, and cutaneous sites of surgical intervention healed properly. No local infections or postoperative deaths occurred as a result of these procedures.

The morphological analysis of the anatomical site treated showed epidural bone formation associated with the biomaterials in the space created between dura mater and internal surface of calvaria 3 month after the surgery (Fig. 2). The histomorphometric analysis of the newly formed tissue in the samples allowed an objective qualitative and quantitative comparison between the sites treated with BCP and β-TCP, respectively, as is shown in Table 1. Measured percentage of new bone in the observed was slightly but significantly higher in the β-TCP 63,6% +/− 8,7 than for BCP 48,5% +/− 9,4. However, the bone seemed to be more mature in BCP augmented sites as indicated by the increased percentage of lamellar bone observed in the (85,7% +/− 13,3) compared to the β-TCP treated group (78,4% +/− 9,5) and smaller percentage of chondroid tissue.

**Fig. 3:**
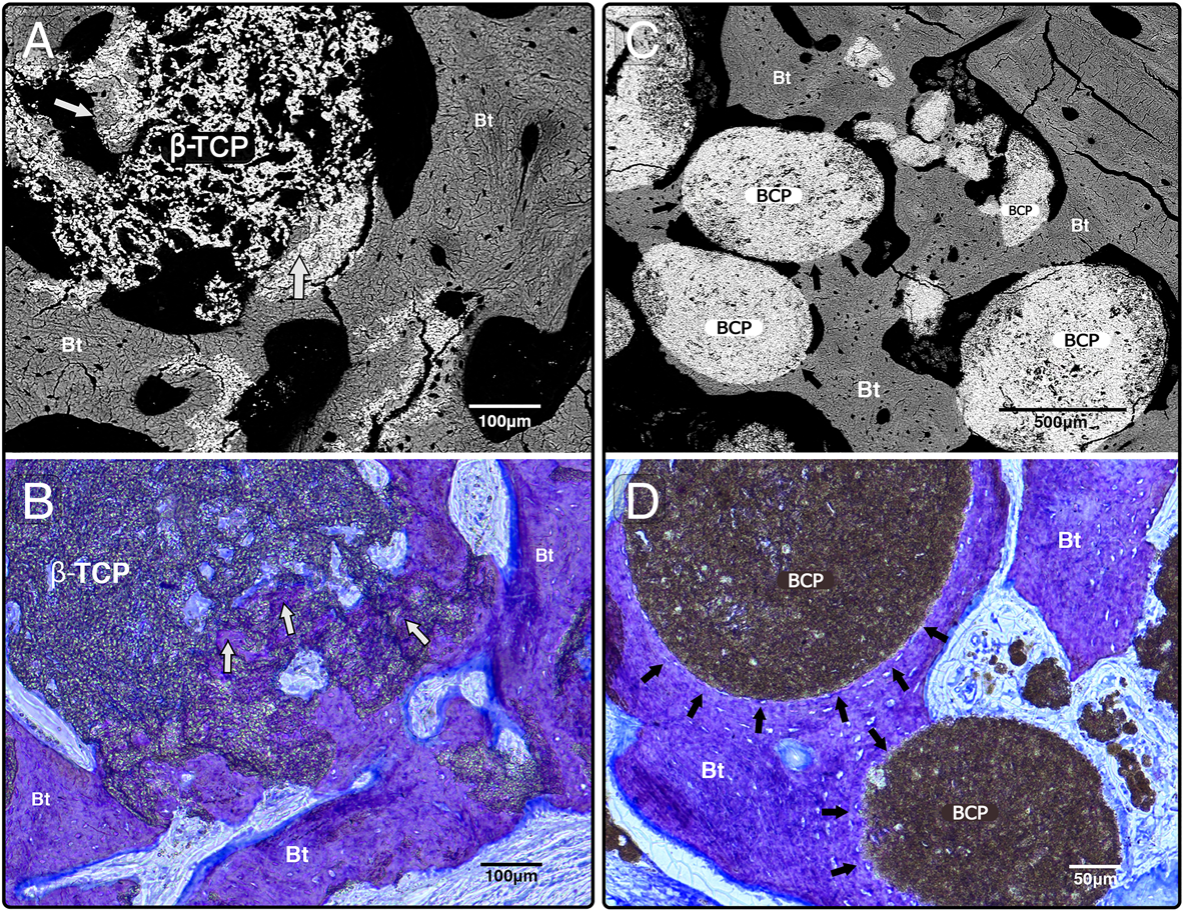
Higher magnification images that show the difference between BCP and β-TCP about new bone formation and osteointegration. In images **a** and **b**, different samples show β-TCP reabsorption and new bone deposit among persistent particles (*white arrows*). Images **c** (BS-SEM) and **d** (histology stain with toluidine blue) show BCP rounded and integrated by bone tissue (*black arrows*). Note in image **d** the osteocytic cells orientation that follows the particle surface form. **a** and **c**, images obtained by BS-SEM; **b** and **d**, histology samples stained with toluidine blue

**Fig. 4:**
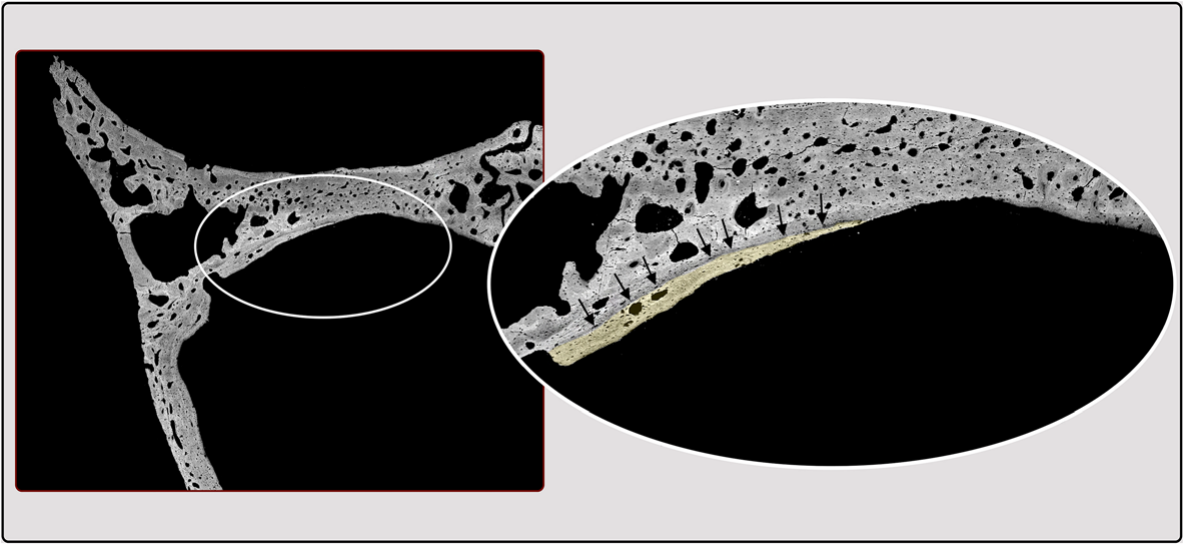
BS-SEM image of a sample obtained from the control specimen #5 (sham operated). In the right side, the area where dura mater was detached from the bone is visible in greater magnification. The thin layer of new bone formed, indicated with black arrows and highlighted with yellow, constitutes a tissue reaction due to the separation of the duramater from the bone surface

In general, the spaces filled with BCP showed structural conservation of particles in close contact with newly formed bone. The sites filled with β-TCP showed degradation of biomaterial with bone tissue formation around and even inside the cracks formed in biomaterial particles (Fig. 3a-b). Comparatively, a higher bone formation was observed area in relation to the β-TCP particles (*p* <0,05). Both biomaterials showed a close relation and integration with bone tissue (Figs. 2e-f and 3c-d). For specimens with unilateral application of biomaterial in the epidural space created (#1 and #2), new bone formation was also observed with similar characteristics to those found in samples with bilateral application of biomaterial (Fig. 2b). In general, at 90 days, the new bone formed showed comparable characteristics of mature trabecular bone with small sectors of fibroreticular bone (woven bone) and chondroid tissue for both biomaterials. In the sham operated sites, the epidural space created was closed, and subperiosteal reaction with scarce bone tissue formation was observed adjacent to the internal cortical surface of the calvarial bone, as shown in Figs. 2a-b and 4.

## Discussion

In our study, the bursa created in the epidural space was maintained when was filled with biomaterial, even with the hydrodynamic intracranial pressure produced by cerebrospinal fluid and the natural meningeal vascularization. While, the bursa created in the sham operated side collapsed and the meninges returned to contact with the intracranial bone surface (Figs. 2a, b and 4). In the experimental zone described, the intracraneal pressure values reported varied from 431.9 to 1250.5 Pa in rabbits from 25 to 42 days in different head position [19]. Other reports in adult rabbits describe 706.6 Pa obtained from the subdural space of the spinal cord [20], and 240.2 Pa obtained from the cistern space of the occipital zone [21].

A permanent pressure on biomaterial inserted in the anatomical site for bone regeneration is a reliable condition in which to observe its properties to maintain the space created while tissue regeneration is in process, a situation that is present, for example, inside the maxillary sinus due to pneumatic pressure changes [1, 22]. This event can influence significantly the sinus floor augmentation, treatment used when there is insufficient bone quantity for dental implants insertion [23].

On the maxillary sinus of rabbit, variations of air pressures of 92.1 Pa (+ − 19.6) in expiration and 95.1 Pa (+ − 39.2) in inspiration [24] have been reported. In human paranasal sinus, the variations of intrasinusal pressure are produced during the final episode of normal expiration and inspiration, with a variation of amplitude of 29.4 – 31.2 Pa, which can increase until 166.7 to 196.1 Pa during physical exercises [25]. Studies in which biomaterials have been tested bilaterally in rabbit maxillary sinuses have shown heterogeneous results [26]. Pneumatic pressure over sinusal mucosa is probably a critical element to consider in this kind of study because this phenomenon may be different for each sinus even in the same specimen, as has been demonstrated in the experiments with occlusion or diameters alteration of the maxillary sinus ostium [1, 22]. This aspect can limit interpretations of comparative studies in the same animal. From this point of view, the epidural model represents a major advance for analysing the biomaterial capacity for maintaining the biological space created, because of the measurable differences found in the intracranial pressures and its parallel with the changing pressure inside the maxillary sinus. Other experimental models for sinus floor augmentation have been developed mainly for larger animals, such as pigs, sheep or dogs due the their anatomical size [27–29]. Nonetheless, the anatomy of these large animal models still differ significantly from the human situation and do have other critical drawbacks such as costs, increased demand for housing space and maintenance, as well as ethical considerations.

Moreover, biomaterials for bone regeneration also are applied in augmentation of alveolar bone inside the mouth and are exposed to various natural local permanent loads, such as the tongue movement or muscular action of lips and cheeks, whose forces are greater and measured in Newtons [30]. Such loads can lead to degradation of newly formed bone and they are difficult to measure and standardise. Consequently, the long-term stability of three-dimensional bone achieved, a determinant factor for bone augmentation success is taken into account in our analysis.

On the other hand, our approach permits a cranial bone defect of a smaller diameter than the usual critical-size and limits the risk of affecting the sutural growing areas [31–33], as is visible in Fig. 2.

In this study, some histologic samples were generated with an *in-situ* hardening BCP and β-TCP applied bilaterally, allowing a more standardized histomorphometric comparison between the results shown for each biomaterial. The metopic suture and the dural septum normally separate the experimental spaces used (Figs. 1 and 2), however both spaces exposed have comparable functional loading conditions, as shown in studies about cerebrospinal fluid hydrodynamic or intracraneal pressure [34]. Consequently, the protocol of this study allows for the control of variables such as the volume of biomaterial inserted and the biological phenomena to which it is exposed during the observation period, contributing to reliable results in terms of qualitative and quantitative comparative analysis of bone regeneration both in cross-sectional and longitudinal studies. In the control sample, where only a bursa in the epidural space was created without placement of biomaterial, scarce bone growth was observed probably due to the physiological reaction after bone-dura mater separation (Figs. 2a-b and 4). These results indicate that the dura mater tends to return to its original position because of the normal intracranial fluid pressure mentioned above. However, despite the described advantages of this animal model, it has a limitation in relation to the small size of the anatomical site: only limited amounts of biomaterials can be applied and tested. For the studies of a major quantity of biomaterial, other models are necessary.

In the absence of bone graft, the dura mater tends to return to its original position contacting the bone surface. In similar form, sinus tends to recover its original shape after sinus lift because of the air pressure [1]. Consequently, the graft behaviour in the site of application is a relevant aspect to consider for maintaining the space created. Autologous bone has been considered as the “gold standard” for grafting the maxillary sinus floor [35] due to it has a higher rate of newly formed bone at the application site when it is compared to other bone substitutes [36, 37]. However, other study showed no significant differences after nine months comparing autologous bone with others grafts considering the total bone volume gained [27, 38]. Also, some disadvantages have been reported about the autogenous bone as an early resorption rate and a greater shrinkage of the elevated sinus floor space when compared with alloplastic materials [39, 40]. A new protocol using epidural space that includes autologous bone is required for contributing to the discussion.

Calcium phosphate is an alloplastic bone reconstruction material that has been used in oral surgery for over 20 years, and it is considered as a bioactive and osteoconductive material [41, 42]. Hydroxyapatite has demonstrated osteoconductive capability, improving the embedding of the biomaterial with the bone structure [43]. In this study, both types of biomaterial, either as particles of pure β-TCP or as BCP combination, showed comparable results in terms of maintenance of the epidural space created. However, the BCP sample appeared to maintain a larger tissue and biomaterial area compared to the β-TCP sample. This observation is in accordance with the resorption characteristics of both biomaterials but needs further confirmation in longitudinal studies with different times of experimental observation.

The content of newly formed bone observed in the specimen with bilateral application of biomaterial was significantly higher with β-TCP compared to the site with BCP (Table 1). This result also could be explained by the different resorption characteristics of β-TCP particles compared to BCP. The β -TCP bonds to bone directly, which granules are rapidly dissolved and newly formed bone fill in the area of dissolved granules [44]. A higher degree of resorption may provide more space inside the β-TCP particles that can be occupied by newly formed bone tissues. Oppositely, the BCP has β-TCP and hydroxyapatite (HA) granules on its composition, observing that HA adheres directly to bone through a collagen-free layer interface with no preferred orientation [44], which is more stable than β-TCP and delaying its resorption. Concerning the samples with BCP, the results showed a close integration between the newly formed bone tissue and the biomaterial (Fig. 3c-d). As depicted in Fig. 3d, the organization of osteocytic lacunae around the particles is compatible with lamellar bone formation. At 90 days of observation, both experimental sites show remnants of the biomaterial particles in contact with osseous trabeculae constituted by woven and lamellar bone and a scarce quantity of chondroid tissue.

Within the limitations of the study, only sham operated sites were used as a control. Furthermore, a small number of rabbits and a single sacrifice period were used. However, the main objective of this study was to describe the first results of the use of epidural space in rabbit to explore the new bone formation associated to biomaterial for bone regeneration in a protected site and subject to permanent hydraulic pressure. Thus, further studies with larger samples, others biomaterials, autogenous bone and more time points could lead to improving the predictability and interpretation of model results.

## Conclusions

The described model to compare the behaviour of two biomaterials for bone regeneration in the bursae created bilaterally in the epidural space provides objective qualitative and quantitative results. The refinement that offers this procedure reduces the number of required experimental animals and produces objective results to compare the osteoconductive potential of biomaterials and its capacity for supporting the permanent physiological forces from cerebrospinal fluid pressure. Using this protocol in association with histology without decalcification also provides reliable comparative histomorphometric analysis.
